# Is the addition of running retraining to best standard care beneficial in runners with medial tibial stress syndrome? Protocol for a randomised controlled trial

**DOI:** 10.1002/jfa2.12029

**Published:** 2024-06-14

**Authors:** Laura M. Anderson, Daniel R. Bonanno, Benjamin J. Calnin, Prasanna Sritharan, Richard W. Willy, Bircan Erbas, Mehak Batra, Hylton B. Menz

**Affiliations:** ^1^ The Injury Clinic South Geelong Victoria Australia; ^2^ Discipline of Podiatry School of Allied Health Human Services and Sport La Trobe University Melbourne Victoria Australia; ^3^ La Trobe Sport and Exercise Medicine Research Centre School of Allied Health Human Services and Sport La Trobe University Melbourne Victoria Australia; ^4^ School of Physical Therapy and Health Sciences University of Montana Missoula Montana USA; ^5^ Department of Public Health School of Psychology and Public Health La Trobe University Bundoora Victoria Australia

**Keywords:** gait retraining, leg injuries, prevention, randomized controlled trial, running injuries

## Abstract

**Background:**

Running retraining is commonly used in the management of medial tibial stress syndrome (MTSS) but evidence for its effectiveness is lacking. The primary aim of this study is to determine if the addition of running retraining to best standard care is beneficial in the management of runners with MTSS.

**Methods:**

This study is an assessor‐blinded and participant‐blinded, parallel‐group, randomised controlled trial. The trial will recruit 64 participants aged between 18 and 45 years, with a clinical diagnosis of MTSS that has affected their running participation for at least four weeks. Participants will be randomised to receive best standard care (control) or running retraining and best standard care (intervention group) over an 8‐week period. Best standard care will consist of load management advice, symptom management advice, footwear advice and a strengthening program. Running retraining will consist of a cue to reduce running step length. Outcomes will be measured at weeks 1, 2, 4 and 8. The primary outcome measure will be the University of Wisconsin Running Injury and Recovery Index at week 4. Secondary outcome measures include: (i) Exercise Induced Leg Pain Questionnaire—British Version, (ii) global rating of change scale, (iii) worst pain experienced during a run, (iv) weekly run volume, (v) reactive strength index score, (vi) single leg hop test, (vii) soleus single leg maximum voluntary isometric contraction, (viii) gastrocnemius single leg maximum voluntary isometric contraction, (ix) single leg plantar flexor endurance test, (x) running step length, and (xi) running step rate. Data will be analysed using the intention‐to‐treat principle.

**Discussion:**

This randomised controlled trial will evaluate if reducing running step length provides additional benefit to best standard care in the management of runners with MTSS over an 8‐week period.

**Trial registration:**

Australian New Zealand Clinical Trials Registry: ACTRN12624000230550.

**Abbreviations**
ATEMPT: adherence to exercise for musculoskeletal pain tool
BMI: body mass index
CV: coefficient of variation
EILPQ‐Br: exercise induced Leg pain questionnaire (British version)
MTSS: medial tibial stress syndrome
MVIC: maximum voluntary isometric contraction
NPRS: numeric pain rating scale
RIR: repetitions in reserve
RPE: rate of perceived exertion
RSI: reactive strength index
UWRI: university of wisconsin running index

## 1 BACKGROUND

Medial tibial stress syndrome (MTSS) is one of the most common running‐related injuries, with an incidence as high as 16% [1, 2]. Medial tibial stress syndrome is typically characterised by exercise‐induced pain along the distal posteromedial border of the tibia [2]. The aetiology of MTSS remains uncertain, with traction periostitis from deep crural fascia and failure of tibial bone remodelling both being suggested as possible causes [2]. As running participation provides many health and social benefits, running‐related injuries such as MTSS can result in reduced or ceased physical activity, incur financial costs, and adversely affect an individual's health.

Considering the prevalence of MTSS, interventions that minimise the reduction of running volume or enable a timelier return to usual running volume are sought by clinicians and the wider running community. There is a lack of evidence for effective interventions for the management of MTSS [2]. Despite this, commonly used treatment strategies include running retraining, lower limb strengthening, rest, stretching, massage, graded running programs, shockwave, and injection‐based therapies [2]. Whilst the evidence regarding effective management of MTSS may be scarce, and with the aetiology remaining uncertain, interventions that reduce tibial loads and improve the capacity of the tissues that attenuate tibial loads are proposed to have a positive effect on symptoms.

Running retraining is advocated by most expert clinicians in the management of MTSS, with strategies intended to reduce impact loading being the most common (e.g., reducing overstride, increasing step rate, transitioning to a midfoot strike) [3]. Several running retraining cues can reduce tibial loads [4, 5, 6, 7], but not all cues provide the same effects [4]. In a probabilistic model, running with a reduced step length decreased the likelihood of tibial stress fracture [5], an injury related to MTSS. To date, evidence to support the use of running retraining for the management of MTSS is lacking as no trials have been conducted to determine clinical effectiveness.

Therefore, the primary aim of this study is to determine if the addition of running retraining, by reducing step length, to best standard care, is beneficial in the management of MTSS in runners.

## 2 METHODS

### 2.1 Study design

The RUNMETRES (RUNning retraining for MEdial Tibial tRESs) study is a parallel‐group randomised controlled trial conducted over an 8‐week period. Participants will be randomised to a control group (best standard care) or an intervention group (best standard care and running retraining). To ensure allocation concealment, permuted block randomisation with random block sizes, stratified by sex at birth will be undertaken using www.sealedenvelope.com. The investigator responsible for recording the outcome measures will be blinded to participants' group allocation. Participants will be blinded to their treatment allocation via limited disclosure, and precautions will be taken to ensure they are unaware of the other treatment group. The trial has been registered on the Australian New Zealand Clinical Trials Registry (ACTRN12624000230550). The findings from the trial will be reported according to the Consolidated Standards of Reporting Trials (CONSORT) statement [6, 7] and the Template for Intervention Description and Replication checklist [8].

### 2.2 Ethical approval

Ethical approval for this study was provided by the Human Studies Ethics Committee at La Trobe University (HEC23408). All participants will provide written informed consent prior to their participation. Ethical standards will adhere to the National Health and Medical Research Council National Statement [9] and the World Medical Association's Declaration of Helsinki [10].

### 2.3 Setting and eligibility criteria

The trial will be conducted at a private community‐based physiotherapy practice (The Injury Clinic, Geelong, Victoria, Australia) and participants will be recruited via:

(i) mail‐out advertisements to local medical and allied health practitioners
(ii) social media advertising campaigns
(iii) posters displayed in local health and fitness related businesses
(iv) contact with local sporting, running and athletic clubs

To be included in the study, participants will be required to meet the following inclusion criteria:

(i) aged 18–45 years
(ii) have a clinical diagnosis of MTSS; according to subjective history and clinical examination [11]:
  ▪ presence of exercise‐induced pain along the tibial periosteum
  ▪ pain provoked by (during or after) physical activity and reduced with relative rest
  ▪ diffuse tenderness on palpation of the tibial periosteum
(iii) have running as a primary exercise activity, participating in running ≥ twice per week
(iv) have a history of MTSS that has affected their running activity for ≥ 4 weeks
(v) have pain consistent with MTSS rated ≥3 out of 10 on a numeric pain rating scale (NPRS)
(vi) be willing to commit to an 8‐week strengthening program that requires completion a minimum of twice per week
(vii) be agreeable to not receive any other forms of treatment for the duration of the trial
(viii) have an ability to understand written and spoken English

Exclusion criteria for participants in this study will include:

(i) any co‐existing lower limb injury in the past 3 months that limits running participation
(ii) an inability to attend the required follow‐up sessions
(iii) the presence of any symptoms suggestive of alternate lower leg pathology inclusive of stress fracture; cramping or burning pain; paraesthesia; severe swelling or erythema
(iv) an inability to run due to the severity of symptoms

### 2.4 Sample size

Sample size was calculated based on the primary outcome measure, the University of Wisconsin Running Injury and Recovery Index (UWRI) [12]. Using a power of 80%, previously reported standard deviation of 6.7 [13], medium effect size (Cohen's *d* = 0.75) of 5 points on the 36‐point UWRI [14] and a significance level set at *α* < 0.05, we estimated using GPower 3.1 (Kiel University, Germany) that a total of 60 participants (i.e., approximately 30 per group) will be required. When estimating sample size we were limited with data available and have opted to use the medium effect size for our estimation. To allow for any imprecision in these estimates, we will have a recruitment target of 32 participants per group.

### 2.5 Interventions

Participants will be randomly allocated to a control group (best standard care) or an intervention group (best standard care and running retraining).

### 2.6 Best standard care

All participants will receive best standard care, consisting of:

#### 2.6.1 Load management advice

Participants will be instructed to ensure a pain level of no more than a 2 out of 10 during a run, reported on a NPRS. At the commencement of the trial an individual run program will be discussed with Laura Margaret Anderson (LMA), with the first 2 weeks not progressing in frequency, volume, or intensity to allow adaptations to the addition of strength training. Following the first two weeks of the trial, weekly running volume will be progressed between 10% and 30% depending on the run training history, time availability, running goals and symptom behaviour of each participant. Weekly running progressions will be programmed and discussed with LMA at week two and week four follow up sessions to ensure an appropriate progression of load for each participant. Participants' running intensity will not be able to exceed a moderate intensity, rated as 6 out of 10 on an 11‐point Borg Scale (0–10) (“labored breathing, challenging and uncomfortable, but sustainable for 30–60 min”) throughout the duration of the trial [15]. Running intensity will be discussed with LMA and details included in weekly run program progressions. If participants experience an exacerbation of their symptoms following a run, they are to report this to LMA and rest until their symptoms have returned to ‘usual’ levels before running again. Their next run is to be completed at an intensity no greater than 4 out of 10 on an 11‐point Borg Scale (“still comfortable, able to hold a conversation”) [15]. If participants experience an exacerbation of their symptoms post‐run over two consecutive run sessions, they are to contact LMA and a modification to their running program will be discussed based on both the severity and time of onset of their symptoms.

The following criteria will be used to guide the progression of each participants running load:

(i) volume will be programmed in minutes; with weekly progressions not exceeding 30%
(ii) intensity will be programmed using rate of perceived exertion (rate of perceived exertion (RPE)) and rated using an 11‐point Borg Scale (0–10) [15]; intensity minutes will be calculated (*RPE  x minutes*) and monitored, with weekly progressions not exceeding 30%
(iii) frequency can be progressed, provided volume progressions do not exceed 30%
(iv) terrain is not to be progressed, and participants are to continue to run on their usual terrain (i.e., surface, elevation etc.)

#### 2.6.2 Symptom management advice

If participants have been using oral or topical medicines (e.g., analgesia, anti‐inflammatories, etc.) to assist with managing their symptoms, they will be advised to continue with their usual use of medicines. If they have not been using medicines in the management of their symptoms, they will be instructed to not commence using medicines to manage their symptoms during the trial. Participants will be requested to document all use of medicines used to manage MTSS symptoms.

Participants will be instructed on the use of a foam roller/release ball on their posterior lower leg muscles. They will be advised to do this a minimum of twice per week, spending approximately 5 minutes on each leg.

#### 2.6.3 Footwear and foot orthoses advice

Participants' running footwear, and foot orthoses if being used for running, will be assessed to determine if appropriate in the opinion of LMA (e.g., running footwear is not showing signs of excessive wear [either on visual observation of the sole of the shoe, or on assessment of foam to ensure no areas of significant collapse]; orthoses have not been recently prescribed, or the participant reports an increase in symptoms or discomfort whilst wearing orthoses). If considered appropriate, participants will be advised to continue to run in the same footwear and foot orthoses for the duration of the trial. If their running footwear is determined to require replacement due to excessive wear, or it is determined to require replacement during the trial, it will be recommended that participants purchase the same footwear model. If the same footwear model cannot be sourced, then a similar replacement model will be recommended following discussion with LMA.

#### 2.6.4 Strengthening program

The strengthening program will be accessible via a custom‐built software (The Injury Clinic Pty Ltd., Geelong, Australia) installed on their phone using a personal login, inclusive of videos and written instructions of the exercises to be performed. Strength programs will be progressed every 2 weeks, however the load of a specific exercise can be progressed within each program according to the prescribed dosages. For exercises to progress, participants will be instructed to ensure a pain level of no more than a 2 out of 10 as rated on an 11‐point NPRS. Participants will be instructed to determine the load of each exercise based on self‐perceived exertion, this will be done through the programming of Repetitions in Reserve (RIR) and, or Rate of Perceived Exertion (RPE). Due to the inclusion of both isometric and isotonic based movements within this program, both RPE and RIR will be used. The program will be structured with exercises in order of priority to complete, and exercises prescribed in groups of two, ‘superset’ together to assist in a timely completion of prescribed exercises. Full details of the strengthening program are provided in Supporting Information S1.

### 2.7 Running retraining

Participants in the intervention group will receive running retraining in addition to best standard care. Running retraining will include a cue to reduce their running step length. Participants will be instructed to shorten their step length by the verbal cue to “land with your foot closer in and under your body”. Compliance with this cue will be assessed with 2D video footage demonstrating a change in lower limb alignment at initial contact. Participants will be issued a Garmin running watch (Garmin Corporation, Olathe, Kansas, USA) that supports the measurement of wrist‐based running dynamics. It will be used to record their preferred running step rate and their step rate when running with the cue for a shorter step length. Although the cue provided to participants will be to “land with your foot closer in and under your body”, the mode of feedback whilst running will be haptic, via the GPS watch, and associated with the step rate recorded when successfully implementing the cue for a shorter step length. For each participant, their Garmin running watch will be setup to provide haptic feedback when they deviate from the target running step rate.

An assessment of participants' running gait will be performed at follow‐up sessions in weeks one, two and four to ensure compliance with the running retraining cue, and they will be provided with real‐time feedback on their running step length and their implementation of the running retraining cue via 2D video footage. Their targeted running step rate (i.e., step rate required to achieve a shortened step length) will be checked and adjusted if needed to ensure the best possible feedback is provided between sessions.

The feedback protocol will be as follows:

*Week 1 and 2* (*acquisition phase*): Haptic feedback will be provided via participant watches during all runs; 2D video feedback will be provided at baseline, week one and week two.

*Week 3 and 4* (*acquisition phase*): Haptic feedback will be provided via participant watches during all runs; 2D video feedback will be provided at week four.

*Week 5 and 6 (transfer phase)*: Intermittent feedback will be provided during runs, with self‐controlled feedback on alternate running sessions.

*Week 7 and 8 (transfer phase)*: No haptic feedback will be provided during runs in the final 2 weeks.

### 2.8 Assessments and data collection sessions

**FIGURE 1:**
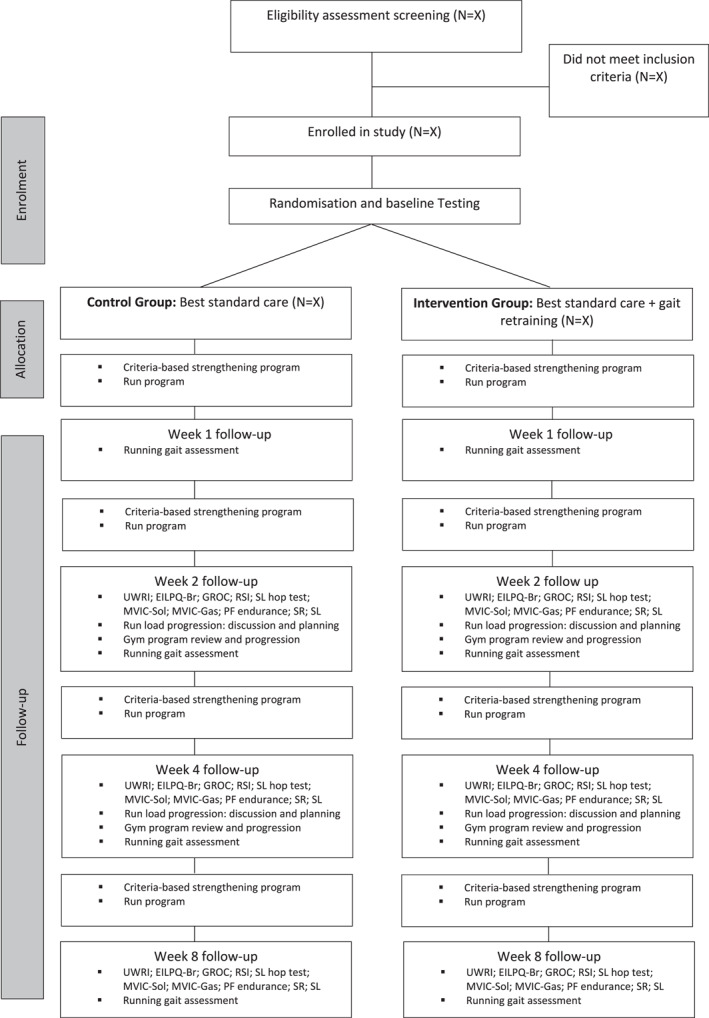
Proposed flow of participants through the trial.

Once participants are determined to be eligible for this study, they will undergo primary and secondary outcome measurements at baseline, 2 weeks, 4 weeks, and 8 weeks. Participants will attend five data collection sessions: session 1 (baseline), session 2 (week one), session 3 (week two), session 4 (week four) and session 5 (week eight). Participants in both groups will receive similar time at each data collection session, with the duration of each session measured for comparison at the end of the trial. Figure 1 outlines the flow of participants through the trial.

### 2.9 Session 1 (baseline)

A clinical examination will be performed to ensure that participants have a clinical diagnosis of MTSS [2]. Participants will complete a questionnaire to obtain information concerning the presentation of their symptoms (lower limb affected, duration of symptoms). Participants' age, sex, gender, height, and mass will be recorded. Data regarding running participation (current running volume, running training history) will also be obtained. During the baseline assessment, participants will have primary and secondary outcome measurements (see ‘outcome measures’ section for details regarding all outcomes) collected prior to being allocated to their intervention.

Participants will receive the first 2 weeks of their strengthening program, with exercises demonstrated by Benjamin James Calnin (BJC). Participants will then be supervised completing each exercise to ensure appropriate technique and safe execution of the program. Instructions regarding the determination of exercise dosage will be given. Participants will be provided with a Garmin running watch for the duration of the trial if they do not own one that meets the needs of the study. Participants in both groups will have 2D video footage of their running collected. Participants allocated to the intervention group will be receive a running retraining intervention to reduce their running step length, and a step rate target for overground running will be determined. The target step rate will be programmed into participants Garmin watch to enable real‐time feedback. No further instructions will be provided to participants allocated to the control group; however, they will be instructed that we will be documenting how their running mechanics change throughout the duration of the trial.

Participants will be instructed on the use of software, as described previously, to record adherence with their strengthening program and how to document their symptom management strategies (e.g., ice, oral medications, etc.). They will also be given further information regarding the secondary outcome measure ‘worst pain experienced during a run’ to be recorded on a NPRS. Participants will discuss their current symptoms and run load with LMA, and an appropriate run program for the first 2 weeks will be outlined.

### 2.10 Session 2 (week one)

At this session, participants in both groups will have their running gait assessed via 2D video. Participants in the intervention group will be provided with real‐time feedback on their running step length. If required, modifications will be made to the step rate feedback for participants in the intervention group to help achieve the desired step length. Participants will also have an opportunity to seek any clarification regarding their strengthening program and symptom management advice.

### 2.11 Session 3 (week two)

At this session, primary and secondary outcome measures will be collected, and participants will be instructed on the next 2 weeks of their running and strengthening program. Each participant will have the progression of their running program reviewed with LMA with any progressions dependent on their current symptoms during and post‐run. Progression of their strength program will depend on their progress and ability to have achieved dosages in their current program, this will be individually discussed with BJC. All participants will have their running gait assessed via 2D video, and participants in the intervention group will be provided with real‐time feedback on their running step length.

### 2.12 Session 4 (week four)

As per session two, participants will have primary and secondary outcome measures collected. Participants will be instructed on the next 4 weeks of progressions of their running and strengthening program. All participants will have their running gait assessed via 2D video, and participants in the intervention group will be provided with real‐time feedback on their running step length.

### 2.13 Session 5 (week eight)

Primary and secondary outcome measurements will be collected, and all participants will have their running gait assessed via 2D video at the conclusion of the trial.

### 2.14 Outcome measures

The primary outcome measure will be the University of Wisconsin Running Injury and Recovery Index (UWRI). The UWRI is a nine‐item running specific patient‐reported outcome measure that relates to both running progression and symptom surveillance. It has been found to have acceptable internal consistency (*α* = 0.82) and test–retest reliability (ICC: 0.93) when assessing running ability following a running‐related injury [12]. Scores range from 0 to 36.

The secondary outcome measures will be as follows.

#### 2.14.1 Exercise Induced Leg Pain Questionnaire—British Version

The Exercise‐Induced Leg Pain Questionnaire (EILPQ‐Br) has been found to have excellent internal consistency (intraclass correlation coefficient [ICC]: 0.92–0.94) and test–retest reliability (ICC: 0.987–0.995) across patients with exercise‐related lower leg pain [16]. The EILPQ‐Br questionnaire comprises 10 items, scored on a five‐point Likert scale from 4 (no difficulty) to 0 (unable to do), there is also a ‘non‐applicable’ category. Scores range from 0 to 40.

#### 2.14.2 Global rating of change scale

Participants will be required to rate their self‐perceived improvement on a 15‐point Global Rating of Change Scale with −7 representing ‘a very great deal worse’ and +7 representing ‘a very great deal better’. Global rating of change scales have been shown to be a valid and reliable way of evaluating self‐assessed clinical progress (ICC 0.90) [17].

#### 2.14.3 Adherence to exercise for musculoskeletal pain tool (ATEMPT)

Participants will be required to rate their adherence to exercise across six items relating to communication with expert; targets; how exercise is prescribed; patient knowledge and understanding; motivation and support; and psychological approach and attitude.

The Adherence to Exercise for Musculoskeletal Pain Tool (ATEMPT) has been shown to be valid and reliable in the measurement of adherence to exercise in musculoskeletal pain presentations [18].

#### 2.14.4 Worst pain experienced during a run—NPRS

Participants will be required to rate their pain on an 11‐point NPRS with 0 representing ‘no pain’ and 10 representing ‘the worst pain imaginable’. The NPRS has been shown to be valid and reliable in measuring changes in both acute and persistent pain states [19]. Participants will record their pain on the NPRS immediately following a run to indicate the worse level of pain they experienced within their run.

#### 2.14.5 Weekly run volume

Participants will record the distance of each run with the use of their Garmin running watch. Total weekly volume for each participant will be downloaded by LMA and documented in an excel spreadsheet (Microsoft Corporation, Washington, USA). Running volume will be recorded as kilometres per week.

#### 2.14.6 Reactive strength index score

Reactive strength index (RSI) is a ratio score that provides a measurement of a person's ability to produce force rapidly and represents an individual's ability to effectively utilise the stretch‐shortening cycle. This typically occurs in movements where body segments are exposed to impact forces that induce stretch (i.e., ground contact when running) [20]. RSI score has been associated with both isometric strength and endurance performance and is calculated by dividing either jump height or flight time by ground contact time and has shown moderate to strong levels of reliability (ICC 0.57—0.99; coefficient of variation 2.98%—14%) across a range of populations [20]. RSI will be assessed on force decks (Force Decks, Vald Performance, Queensland, Australia) using a drop jump to a standardised height of 30 cm [20], and the measurement taken from flight time and contact time data. Participants will initially stand with one foot on each force deck to allow calibration, they will then be instructed to stand on a 30 cm box placed behind the force decks, keeping their posture upright and their eyes forward. When ready, they will be instructed to step off the box (stepping out and not down) to ensure the drop height remains 30 cm, landing on both legs at the same time before immediately jumping as high as possible with the shortest possible contact time with the ground. RSI scores will be calculated using the following equation:

$$Reactive\;strength\;index\;\left(RSI\right)=\frac{Flight\;time\;\left(ms\right)}{Contact\;time\;\left(ms\right)}$$

#### 2.14.7 Single leg hop test

A single leg repetitive hop test will be used to obtain a measure of vertical stiffness for both limbs. The testing of repeated single leg vertical hops measured on portable force plates has been shown to be reliable in a healthy athletic population (ICC ≥0.97) [21]. Participants will be instructed to stand on force decks (Force Decks, Vald Performance, Queensland, Australia) whilst they are calibrated. They will then be instructed to place their hands on their hips and hop continuously on one leg whilst minimising contact time for 10 hops. The hopping test will be performed on both left and right sides. RSI scores will be calculated from the hop flight and contact times as previously described, and data from the best five hops on each leg will be used.

#### 2.14.8 Maximum voluntary isometric contraction—soleus (single leg)

Previous research has found symptomatic runners with MTSS to have reduced ankle plantar flexor muscle endurance [22, 23]. Both studies evaluated plantar flexor muscle endurance measures with a standing heel‐raise test. Mattock and colleagues also assessed maximal voluntary isometric contraction (MVIC) strength of flexor hallucis longus, flexor digitorum, soleus, peroneal tibialis anterior and soleus muscle, measured with a hand‐held dynamometer and found significant strength deficits in flexor hallucis longus, peroneal, soleus and tibialis anterior [23]. We will be testing MVIC with the use of force decks (Vald Performance, Queensland, Australia). Use of force deck to test the isometric strength of soleus has been reported to be reliable in a population of elite footballers [24]. Participants will be seated in a position that ensures a testing bar height with participant's hip and knee at 90° flexion. The foot being tested will be placed in the centre of the force plate and in a position that has the ankle at 90° dorsiflexion. Force decks will then be calibrated with participants in the appropriate testing position. Participants will be advised to maintain a neutral foot position and apply minimal pre‐tension on the bar until the verbal instruction is given to commence the test. Before each repetition, the participant will be guided by a countdown (“3, 2, 1”) and instructed to push for 3 seconds up and against the bar as hard and as fast as possible [24]. Three repetitions will be conducted, with data representing the best of the three trials. Maximal voluntary isometric contraction will be represented as peak force in newtons per kilogramme.

#### 2.14.9 Maximum voluntary isometric contraction—gastrocnemius (single leg)

Following warm‐up and familiarisation, participants will be instructed to stand with their tested leg in the centre of the force deck (Force Decks, Vald Performance, Queensland, Australia), in this position force decks will be calibrated prior to commencing testing. Bar height will be determined to ensure participants knee is in an extended position (0° flexion), this will be confirmed with a goniometer. Foam padding (Iron Edge, Melbourne, Australia) will be placed around the testing bar to ensure comfort on participants' shoulders and upper back during the test. Participants will be advised to apply minimal pre‐tension on the bar until the verbal instruction is given to commence the test. Before each repetition, the participant will be guided by a countdown (“3, 2, 1”) and instructed to push for 3 seconds up and against the bar as hard and as fast as possible [24]. Three repetitions will be conducted, with data representing the best of the three trials. Maximal voluntary isometric contraction will be represented as peak force in Newtons per kilogram.

#### 2.14.10 Plantar flexor endurance test (single leg)

Plantar flexor endurance will be measured using the standing heel‐rise test. This test has been shown to be reliable in healthy populations (ICC 0.79—0.96), but to our knowledge has not been assessed for reliability in people with MTSS [22]. Following warm‐up and familiarisation, participants will be required to perform as many single leg calf raises as possible. Participants will start standing with their forefoot on the edge of a box, with index and middle fingertip support from both hands on the wall at shoulder height for balance permitted. They will be required to maintain the knee of their tested leg straight (extended) during testing. Participants will be asked to stand on one foot and raise the heel of their ipsilateral limb as high as possible so a string line can be placed contacting the proximal dorsal aspect of the foot to identify the target height of a complete repetition. Participants will then be instructed to raise and lower their heel to the beat of a 60 Hz metronome, lifting their heel as high as possible to contact the string line in one beat and lowering it in one beat (i.e., 30 calf raise repetitions per minute). Participants will be encouraged to go through the full range of available motion. The test will be terminated once participants are no longer able to perform a repetition, cannot maintain the beat of the metronome, demonstrate compensatory movements (e.g., knee flexion, trunk lean, hip strategy), or show marked reduction in heel range of motion. Two minutes rest will be allocated between legs [25]. Scores will be represented as number of single leg calf raises.

#### 2.14.11 Step rate

Step rate during running will be recorded using Garmin running watch that support the measurement of wrist‐based running dynamics. Measurements of step rate with Garmin running watches have been shown to be valid and reliable (ICC = 0.931) [26]. In addition, participants' running step rate will be manually counted and recorded over four separate 30‐s intervals during follow‐up sessions. Step rate will be recorded as number of steps per minute.

#### 2.14.12 Step length

Step length during running will be calculated from running step rate data provided from each participants Garmin running watch using the following equation: *velocity (m/min) ÷ step rate*.

$$Step\;length\;\left(m\right)=\frac{Run\;speed\;\left(m/\min\right)}{Step\;rate\;\left(steps/\min\right)}$$

Data will be recorded throughout the trial, and measurements recorded and correlated to 2D video footage at baseline, one, two, four and 8 weeks. Step length will be recorded in metres.

### 2.15 EVALUATION OF INTERVENTION ADHERENCE

Adherence to interventions and training load will be documented throughout the trial. Participants will be required to record their adherence to the strength program, symptom management advice, response to their run sessions, and run volume via a custom‐built software (The Injury Clinic Pty Ltd., Geelong, Australia) installed on their phone. Adherence to a reduction in running step length will be confirmed at sessions 1 to 5, and the step rate required to achieve the target step length will be recorded by the Garmin running watch for each run. Adherence will be considered to be acceptable if participants in both the intervention and control groups complete 80% of their programmed strength sessions, and if participants in the intervention group complete 80% of their running retraining sessions [27]. Participants strength program will be considered complete if a minimum of 8 out of 12 exercises have been completed within each session.

### 2.16 Adverse events

Complications and adverse events associated with the intervention will be recorded in participant files and reported in the final manuscript. Participants will be asked to document the type of adverse event, the body location, the frequency and/or severity of the event. An adverse event will be considered any harmful or unpleasant outcome for which there is a known or plausible association with the interventions and those for which there is none [28].

## 3 DATA ANALYSIS

### 3.1 Descriptive analysis

Differences between the control and intervention group in demographic and clinical characteristics and for the primary and secondary outcome measures (2, 4, and 8 weeks) will be described and assessed using *t* tests for continuous and chi‐square for categorical variables. If after assessment of the assumptions of the *t* test (such as the homogeneity of variance) are violated, we will use alternatives such as the Welch's test with Satterhwaite approximation as a sensitivity analysis. For cell counts <5, *p* values from chi‐square tests will not be reported.

### 3.2 Mixed effects analysis

A mixed effects model will be used to assess the mean differences between the groups for the outcomes considered. The fixed effects are between‐participant variables such as age, sex, BMI, symptom management and footwear. These models incorporate time as the random effects which is a within‐subject variable. All assumptions for mixed effects modelling will be checked. As most of the outcome measures will be considered as continuous, normality and homogeneity of variance assumptions will be assessed. If these assumptions are violated, alternative modelling approaches will be considered. Effect modification by age, BMI and sex will be explored using likelihood ratio tests, taking into consideration the sample sizes of the subgroups. Strata‐specific results will be reported if the *p* value for the interaction terms is < 0.1, and if at least one of the observed associations from the strata‐specific models are statistically significant at *p* ≤ 0.05. Results from these models will be presented as estimated effect sizes (both unadjusted and adjusted), standard errors, 95% confidence intervals and *p* values. All statistical analyses will be performed using the latest version of Stata (StataCorp, College Station,TX, USA).

## 4 DISCUSSION

This is the first randomised controlled trial to determine the effectiveness of running retraining in the management of MTSS in runners. Although laboratory‐based studies have previously shown that running retraining strategies can be used to decrease tibial loads [29, 30, 31], there is a lack of evidence for whether it provides improvements in pain and function for runners with MTSS.

The running retraining intervention (cueing a shorter step length) and its implementation was selected for this trial for three main reasons. Firstly, a relationship between reducing running step length and reducing tibial load and the probability of a tibial bone stress injury [5] has been well reported [5, 31], and the consequence of increasing the number of loading cycles by increasing the number of steps does not appear to increase injury risk [5]. Research has also indicated that kinematic factors such as the orientation of the hip, knee and ankle for a given stride length may be critical when considering the magnitude of tibial acceleration [31]. Secondly, despite the widespread understanding of an inverse relationship between decreasing step length and increasing step rate, data from our previous study indicates differences in the implementation of these cues, with a reduction in step length having a preferred effect on tibial loads at both a comfortable and moderate running pace [4]. Thirdly, the implementation of the running retraining strategy is designed to provide participants with adequate feedback to retrain their running gait, including both acquisition and transfer phases to assist with motor skill learning [32]. This will be achieved via real‐time haptic feedback from a Garmin running watch that monitors step rate while running, and verbal and visual feedback via 2D video footage at various timepoints in the trial. As the selected running watch is commercially available and widely used by runners, it should enable our running retraining intervention and its implementation to be readily utilised in the management of MTSS in runners if it is found to be effective in this trial.

Previous studies investigating running retraining to reduce tibial loads have found real‐time visual feedback [29, 30], haptic feedback [33], and clinician verbal feedback [29] are all effective in reducing tibial loads. Whilst these studies have demonstrated that running retraining can be effective in reducing tibial loads, none have examined the clinical effectiveness of running retraining among runners with MTSS. In addition, there is an absence of studies investigating the effects of running retraining on tibial loads beyond immediate effects. Despite this, running retraining is a common strategy adopted by clinicians and coaches in the management of running related lower‐leg pain [3]. With few running retraining trials conducted for running‐related injuries, the expected rates and types of adverse events remain somewhat unknown. However, as the cue to reduce step length will likely result in minimal changes to other gait parameters [4] the risk of adverse events is considered negligible.

Whilst ‘best standard care’ for MTSS has not yet been established, a clinical trial by Mendez‐Rebolledo and colleagues found that the inclusion of 6 weeks of neuromuscular training (plyometric and weightbearing drills) in uninjured female athletes reduced the incidence of MTSS [34]. To our knowledge, the effect of a strength training program inclusive of plyometric exercises on runners with MTSS has yet to be investigated. The inclusion of ‘best standard care’ as a comparator intervention assists with accounting for non‐intervention effects (e.g., placebo effect, Hawthorne effect, natural resolution, etc.) not directly related to the running retraining intervention. Providing participants with an intervention that they perceive as being equally credible and expected to provide similar benefits as the experimental intervention will assist in minimising confounding factors such as resentful demoralisation among participants not receiving the running retraining. As all participants will spend a similar amount of time with clinicians, receive the same number of appointments, their experience in the trial is expected to be very similar (with the only exception being the inclusion of running retraining).

The eligibility criteria do not require participants to have a particular running gait pattern. Although running retraining is most likely used in a clinical setting to change a gait parameter believed to be associated with MTSS, it is not currently possible to identify individuals with MTSS most likely to benefit from running retraining. In addition, decreasing step length has been shown to reduce tibial loads in uninjured runners with no pre‐specified gait characteristics, for example, a long baseline step length [4]. Since a specific gait parameter is not an inclusion criterion, it is possible that not all participants included in our trial will potentially experience a reduction in tibial loads by reducing their step length. Although any expected improvements in pain and function is proposed to result from a reduction in tibial load, it is also possible that a clinical benefit may result from another mechanism of action related to a reduction in step length that currently remains unknown.

This trial has been designed to optimise its scientific rigour while ensuring a high clinical utility. Some strengths of the trial include the use of allocation concealment, blinded data entry, adhering to the intention‐to‐treat principle to analyse data, and the use of a comparator intervention as a control. In addition, this trial has been designed to have high external validity, with the eligibility criteria being representative of a demographic most likely to experience MTSS, and the study being conducted in a real‐world setting, ensuring any findings regarding the success of interventions can be transferable to clinical practice. If effective, the gait retraining program's use of a commercially available wearable device ensures that our findings will be reproducible and will translate readily to clinical settings. However, due to the nature of the interventions, inherent limitations with this trial are a lack of blinding of the primary investigator and blinding of participants via limited disclosure. We acknowledge the potential for clinician bias, and plan to address this by ensuring a similar amount of time is spent with each participant, that the duration of each data collection session be recorded to allow a comparison at the conclusion of the trial, and that an assessor blinded to group allocation be involved in all decision making regarding best standard care and progression of load. Accordingly, the findings of this trial will need to be viewed in consideration of its strengths and limitations.

## 5 CONCLUSION

This randomised controlled trial will determine if running retraining to reduce step length is beneficial in the management of runners with MTSS. Specifically, it will determine whether reducing running step length provides additional benefit to best standard care over an 8‐week period.

## AUTHOR CONTRIBUTIONS

**Laura Anderson**: Conceptualisation; methodology; visualisation; writing – original draft; writing – review and editing. **Daniel Bonanno**: Conceptualisation; methodology; supervision; visualisation; writing – original draft; writing – review and editing. **Benjamin Calnin**: Methodology; visualisation; writing – review and editing. **Prasanna Sritharan**: Software; writing – review and editing. **Richard Willy**: Methodology; supervision; writing – review and editing. **Bircan Erbas**: Methodology; supervision; writing ‐ review and editing. **Mehak Batra**: writing – review and editing. **Hylton Menz**: Conceptualisation; methodology; supervision; visualisation; writing – original draft; writing – review and editing.

## CONFLICT OF INTEREST STATEMENT

All authors declare that they have no competing interests.

## ETHICS STATEMENT

Ethical approval for this study was provided by the Human Studies Ethics Committee at La Trobe University (HEC23408). All participants will provide written and verbal informed consent prior to their participation.

## CONSENT FOR PUBLICATION

Not applicable.

## Supporting information

Supporting Information S1

## Data Availability

The data that support the findings of this study are available on request from the corresponding author. The data are not publicly available due to privacy or ethical restrictions.
