# Supplementary material for: Is the addition of running retraining to best standard care beneficial in runners with medial tibial stress syndrome? Protocol for a randomised controlled trial
Source: J Foot Ankle Res. 2024 Jun 14;17(2):e12029. doi: 10.1002/jfa2.12029 (PMC11296717; doi:10.1002/jfa2.12029)
Supplement: Supplementary file 1 — Supporting Information S1 [file JFA2-17-e12029-s001.docx]

**Additional File 1: Strengthening Program**

**Table 1: Participants strengthening program set out into weekly progressions, with exercises categorised based on their intended target**

| **Week** | **Lower Limb Strength Capacity** | **Global Strength** | **Lumbo-Pelvic Control** | **Coordination & Patterning** |
| --- | --- | --- | --- | --- |
| 1-2 | Seated Calf Isometric  3x30sec; RPE 7  Single Leg Standing Calf Isometric 3x30sec; RPE 7  Tibialis Posterior Isometric (Overcoming)  3x20sec; RPE 7 | BB Hip Thrust 3x12; 2 RIR  SL RDL 3x15; 2 RIR  Goblet Squat 3x15; 2 RIR  Drop Landing 3x8 | Side Plank 3x30sec; RPE 9  Banded Hip Abduction 3x20ea; 1 RIR | Wall March 3x20  Penguin Walks 3x20sec  Assisted Pogo 3x20 |
| 2-4 | Seated Calf Raise  3x12; 2 RIR  Single Leg Standing Calf Raise 3x12; 2 RIR  Tibialis Posterior Isometric (Yielding)  3x20sec; RPE 7 | SL Hip Thrust 3x12ea; 2 RIR  SL RDL 3x12ea; 2 RIR  RFESS 3x12ea; 2 RIR  SL Drop Landing 3x5ea | Side Plank 3x1min each; RPE 9  Cable Hip Abduction 3x12; 2 RIR | Wall Switch 3x20  Rudiment Hops 3x20sec ea  Pogos (Low Amplitude) 3x15 |
| 4-6 | SL Seated Calf Isometric  3x30sec; RPE 8  SL Standing Calf Raise  3x8; RPE 8  Tibialis Posterior Isometric (Oscillating)  3x20; RPE 7 | BB Hip Thrust 3x8; 2 RIR  SL RDL 3x10ea; 2 RIR  RFESS 3x10ea; 2 RIR  Tuck Jump (Single) 3x8 | SL Side Plank 3x20sec each; RPE 9 | Sled Marching 3x30sec  Pogos (Extensive) 3x20sec  Hurdle Hop (Recovery Hop) 3x10 |
| 6-8 | SL Seated Calf Raise  3x10ea; RPE 8  SL Standing Calf Raise (Eccentric Overload)  3x8ea; RPE 8  Tibialis Posterior Isometric (Oscillating)  3x20; RPE 7 | BB Hip Thrust 3x8; 2 RIR  SDL RDL 3x10ea; 2 RIR  RFESS 3x10ea; 2 RIR  Tuck Jump (Reactive Hop) 3x8 | SL Side Plank 3x30sec each; RPE 9 | Sled Marching (Added Intent) 3x30sec  Pogos (Extensive) 3x20sec  SL Hurdle Hops (Recovery Hop) 3x5ea |

(Abbreviations: RPE = rate of perceived exertion; RIR = repetitions in reserve; BB = barbell; SL = single leg; RDL = romanian deadlift; RFESS = rearfoot elevated split squat)

**Table 2: Participant view of strength program for weeks one and two**

| **GROUP** | **EXERCISE** | **DOSAGE** |
| --- | --- | --- |
| 1A | Wall March | 3 x 20 |
| 1B | Penguin Walk | 3 x 20sec |
| 2A | Assisted Pogos | 3 x 20 |
| 2B | Drop Landing | 3 x 8 |
|  | | |
| A1 | Hip Thrust | 3x12; 2 RIR |
| A2 | Seated Calf Isometric | 3x30sec; RPE 7 |
|  | | |
| B1 | SL RDL | 3x15; 2 RIR |
| B2 | SL Standing Calf Isometric | 3x30sec; RPE 7 |
|  | | |
| C1 | Goblet Squat | 3x15; 2 RIR |
| C2 | Side Plank | 3x30sec; RPE 9 |
|  | | |
| D1 | Tib Post Isometric (Overcoming) | 3x20sec; RPE 7 |
| D2 | Banded Hip Abduction | 3x20ea; 1 RIR |

**Table 3: Participant view of strength program for weeks 3 and 4**

| **GROUP** | **EXERCISE** | **DOSAGE** |
| --- | --- | --- |
| 1A | Wall Switch | 3 x 20 |
| 1B | Rudiment Hops | 3 x 20sec ea |
| 2A | Pogos | 3 x 15 |
| 2B | SL Drop Landing | 3 x 5 ea |
|  | | |
| A1 | SL Hip Thrust | 3 x 12 ea; 2 RIR |
| A2 | Seated Calf Raise | 3 x 12; RPE 7 |
|  | | |
| B1 | SL RDL (remove hand assist) - | 3 x 12; 2 RIR |
| B2 | SL Standing Calf Raise (5 SEC ECC) | 3 x 12 ea; RPE 7 |
|  | | |
| C1 | RFESS | 3 x 12 ea; 2 RIR |
| C2 | Side Plank | 3 x 1min ea; RPE 9 |
|  | | |
| D1 | Tib Post Isometric (Yielding) | 3 x 20sec; RPE 7 |
| D2 | Cable Hip Abduction | 3 x 12 ea; 2 RIR |

**Table 4: Participant view of strength program for weeks 5 and 6**

| **GROUP** | **EXERCISE** | **DOSAGE** |
| --- | --- | --- |
| 1A | Sled Marching | 3 x 30 sec |
| 1B | Pogos (Extensive) | 3 x 20 sec ea |
| 2A | Tuck Jump (Single) | 3 x 8 |
| 2B | Hurdle Hops (Recovery Hop) | 3 x 10 |
|  | | |
| A1 | Barbell Hip Thrust | 3 x 8; 2 RIR |
| A2 | SL Seated Calf Isometric | 3 x 30sec ea; RPE 8 |
|  | | |
| B1 | SL RDL | 3 x 10; 2 RIR |
| B2 | SL Standing Calf Raise | 3 x 8ea; RPE 8 |
|  | | |
| C1 | RFESS | 3 x10 ea; 2 RIR |
| C2 | SL Side Plank | 3 x 20-30 sec ea; RPE 9 |
|  | | |
| D1 | Tib Post Isometric (Oscillating) | 3 x 20 sec; RPE 7 |
| D2 | Cable Hip Abduction | 3 x 20 ea; 1 RIR |

**Table 5: Participant view of strength program for weeks 7 and 8**

| **GROUP** | **EXERCISE** | **DOSAGE** |
| --- | --- | --- |
| 1A | Sled Marching (Added Intent) | 3 x 30 sec |
| 1B | Pogos (Extensive) | 3 x 20 sec ea |
| 2A | Tuck Jump (Reactive Hop) | 3 x 8 |
| 2B | SL Hurdle Hops (Recovery Hop) | 3 x 10 |
|  | | |
| A1 | Barbell Hip Thrust | 3 x 8; 2 RIR |
| A2 | SL Seated Calf Raise | 3 x 10 ea; RPE 8 |
|  | | |
| B1 | SL RDL | 3 x 10; 2 RIR |
| B2 | SL Standing Calf Raise (Eccentric Overload - Up 2 Down 1) | 3 x 8 ea; RPE 8 |
|  | | |
| C1 | RFESS | 3 x 10ea; 2 RIR |
| C2 | SL Side Plank | 3 x 45sec ea; RPE 9 |
|  | | |
| D1 | Tib Post Isometric (Oscillating) | 3 x 20 sec; RPE 7 |
| D2 | Cable Hip Abduction | 3 x 20 ea; 1 RIR |
